# Effects of atmospherically relevant PM_2.5_ on skeletal muscle mitochondria: a review of damage mechanisms and potential of exercise interventions

**DOI:** 10.3389/fpubh.2025.1615363

**Published:** 2025-06-04

**Authors:** Yi Ding, Qiliang Wan, Wenduo Liu

**Affiliations:** Department of Sports Science, College of Natural Science, Jeonbuk National University, Jeonju, Republic of Korea

**Keywords:** particulate matter, skeletal muscle, mitochondrial function, exercise, fine dust

## Abstract

This study aims to explore the multifaceted impacts and mechanisms of fine particulate matter (PM_2.5_) exposure on skeletal muscle mitochondria. Evidence suggests that PM_2.5_ can penetrate the respiratory barrier and enter the circulatory system, spreading throughout the body and causing significant damage to the morphology, quantity, and function of skeletal muscle mitochondria. This is manifested by a decline in oxidative phosphorylation efficiency and mitochondrial dysfunction. Meanwhile, PM_2.5_ exposure induces excessive production of reactive oxygen species, triggering oxidative stress responses that impair mitochondrial dynamic regulation. This further disrupts the balance of glucose and lipid metabolism in skeletal muscle, exacerbating the development of metabolic diseases. The review underscores the systemic effects on skeletal muscle following mitochondrial dysfunction after PM_2.5_ exposure and the preventive and treatment potential of exercise.

## 1 Introduction

With the rapid economic development and continuous industrial expansion, the increase in particulate matter (PM) in the air has become increasingly severe (1), making particulate pollution a significant global issue (2). In the 2006 World Health Organization Air Quality Guidelines, PM was recommended as an indicator of particulate pollution in the air (3), representing a highly complex mixture of particles with varying sizes in the air (4), and typically categorized based on aerodynamic diameter into PM_10_ (2.5–10 μm) and PM_2.5_ (<2.5 μm) (5). PM_2.5_ penetrates deeply into the alveoli and can remain in the lungs (6). These particles may also cross the air–blood barrier, enter the bloodstream, and trigger systemic inflammation (7, 8). Furthermore, acute inhalation of high concentrations of PM_2.5_ can also impair athletic performance (9).

It has been reported that toxic substances and heavy metals in PM_2.5_ particles can induce excessive production of reactive oxygen species (ROS), thereby triggering oxidative stress responses in the body (10, 11). Under oxidative stress conditions, ROS disrupt normal cellular physiological pathways (11, 12). Furthermore, due to the microscopic characteristics of PM_2.5_ (13), it can penetrate cell membranes and enter mitochondria (14), leading to insufficient adenosine triphosphate (ATP) supply to meet cellular metabolic demands, thereby causing energy metabolism disorders (15). This disruption further contributes to chronic inflammation and insulin resistance (16, 17), ultimately increasing the incidence of diabetes and metabolic diseases (18, 19). In addition, PM_2.5_ exposure also causes severe damage to various tissues, including the lungs (20), liver (21), adipose tissue (22), heart (23), and skeletal muscle (24).

The aforementioned evidence indicates that PM_2.5_ exposure not only disrupts systemic metabolic homeostasis but may also exert profound effects on key metabolic organs. It is well known that skeletal muscle is the largest metabolic organ in the human body (25, 26), not only playing a crucial role in regulating overall metabolism (27), but also in maintaining glucose and lipid metabolic homeostasis (28), utilizing various metabolic pathways to ensure the stability and efficiency of energy supply within the body (29). Compared to other tissues, skeletal muscle has a high density of mitochondria and a dense network of capillaries, which makes it highly dependent on respiration and oxygen exchange (30). Previous studies have shown that PM_2.5_ inhaled through the lungs can increase oxidative damage levels in peripheral blood (31). Due to its high blood flow perfusion and active material exchange (32), skeletal muscle may become one of the primary target tissues for PM_2.5_ accumulation and damage. These physiological characteristics determine the vulnerability of skeletal muscle to PM_2.5_ exposure.

PM_2.5_ exposure leads to excessive ROS production (10), disrupting the redox balance in myocytes and resulting in insufficient energy supply (33). Since mitochondria are the “central regulators” of cellular energy metabolism (34, 35), the negative reactions induced by PM_2.5_ exposure may not only damage cells but also impair mitochondrial function (36). Under normal conditions, mitochondria provide substrates (NADH and FADH2) for ATP synthesis to oxidative phosphorylation (OXPHOS) through the tricarboxylic acid (TCA) cycle (37), followed by the transfer of electrons through the electron transport chain (ETC), creating a proton gradient (38). Ultimately, ATP is synthesized via ATP synthase (39). This entire process efficiently converts glucose and fatty acids into ATP (40). Moreover, it can be flexibly adjusted based on cellular environment and energy demands, providing a stable energy source for skeletal muscle (41). PM_2.5_ exposure inhibits the expression of citrate synthase activity in skeletal muscle mitochondria (42), thereby reducing the generation of TCA cycle substrates (NADH and FADH2) and ultimately leading to a decline in ATP production efficiency (43). Furthermore, an experimental study observed that short-term PM_2.5_ exposure causes damage to mitochondrial morphology and dynamics in skeletal muscle, leading to the disruption of mitochondrial homeostasis (24).

In summary, the impact of PM_2.5_ exposure on skeletal muscle mitochondria is profound, not only damaging mitochondrial morphology but also significantly affecting their functionality and metabolic capacity. The existing research on the effects of PM_2.5_ exposure on skeletal muscle mitochondria lacks a systematic perspective. Therefore, this review aims to explore the effects and mechanisms of PM_2.5_ exposure on mitochondrial morphology and function in skeletal muscle, as well as the potential threats these issues pose to the skeletal muscle system. For this purpose, this study included “original research conducted under PM_2.5_ particle exposure models,” excluding other pollutants or finer particles (such as PM_0.1_). The study subjects were limited to rodents or humans. The literature screening period was from 2000 to 2025. This work seeks to provide new insights and references for the prevention and treatment of related diseases in the context of contemporary environmental PM_2.5_ pollution.

## 2 The impact of PM_2.5_ exposure on skeletal muscle mitochondria

Studies have shown that PM_2.5_ exposure has long-term damaging effects on skeletal muscle mitochondria, including morphological damage, inhibition of mitochondrial biogenesis-related pathways, reduced mitochondrial enzyme expression levels and mitochondrial function. Table 1 summarizes studies on the effects of PM_2.5_ exposure on skeletal muscle mitochondria.

**Table 1:** Effects of PM_2.5_ exposure on skeletal muscle mitochondria.

Studies	Treatment (PM)	Key findings	References
Mouse on days 10 of gestation	50 μL of particle suspension via oropharyngeal aspiration daily for 7 days (atmospheric source PM_2.5_)	Skeletal muscle mitochondrial DNA copy number, lower mRNA levels of electron transport genes and reduced citrate synthase activity in offspring mouse	Stephenson et al. (42)
8-week-old mouse	100 μg/m^3^, 1.5 h/day, 7 days (atmospheric source PM_2.5_)	Hydrogen peroxide generation and mitophagy level were significantly increased. Mitochondrial DNA level and cytochrome c oxidase activity were significantly decreased	Park et al. (126)
7-week-old mouse	71.20 ± 45.01 μg/m^3^, 8 h/day, 2/4/6 months (atmospheric source PM_2.5_)	The mitochondrial damage level and mRNA expression of the dynamics related factors DRP-1, FIS-1, MFN-1/2 and OPA-1 increased simultaneously. SIRT-1, AMPKα, PGC-1α, and NRF-1 protein expression levels were significantly decreased	Fan et al. (72)
16-week-old mouse	50.1 ± 8.1 μg/m^3^, 2 h/day, 5 days (atmospherically relevant artificial PM_2.5_)	The mitochondrial damage level was significantly increased. MFN-1, PGC-1α, SDHB and COX-1 protein expression levels were significantly decreased	Liu et al. (24)
4-week-old mouse	50.9 ± 10.4 μg/m^3^, 2 h/day, 5 days (atmospherically relevant artificial PM_2.5_)	The mitochondrial damage level was significantly increased. PGC-1α, NADH-UO, SDHB, COX-1 and COX-4 protein expression levels were significantly decreased	Liu et al. (71)

### 2.1 Effects of PM_2.5_ exposure on mitochondrial morphology

Skeletal muscle mitochondria possess a unique double-membrane structure (44). The outer mitochondrial membrane (OMM) faces the cytoplasm, serving as a barrier and protective layer, while the inner mitochondrial membrane (IMM) is highly folded into cristae, forming a complex sac-like structure (45, 46). This unique architecture plays a pivotal role in energy production processes (47). The mitochondrial cristae are enriched with complexes required for the electron transport chain and oxidative phosphorylation (48). The extensive folding of the cristae significantly increases the surface area of the inner membrane, thereby enhancing the number of attachment sites for these complexes (49, 50). This structural adaptation facilitates the efficiency of OXPHOS, providing an optimal environment for ATP synthesis (51). The mitochondrial membranes ensure the functional integrity of mitochondria (52). Additionally, mitochondria exhibit dynamic properties by regulating their size and quantity through the processes of fission and fusion, allowing them to adapt to cellular energy demands (53, 54). This dynamic nature relies on the coordinated interplay of biogenesis, autophagy, and mitochondrial dynamics (55), forming a homeostatic network within the cell (56). The regulation of mitochondrial biogenesis primarily depends on a series of critical factors (57, 58). Among these, peroxisome proliferator-activated receptor gamma coactivator 1-alpha (PGC-1α) serves as the primary driver of mitochondrial biogenesis (59–61). PGC-1α upregulates the expression of nuclear respiratory factor 1 (NRF-1), which subsequently activates mitochondrial transcription factor A (mTFA), promoting the replication and transcription of mitochondrial DNA. This process increases mitochondrial quantity and quality to meet cellular energy demands (62–64). Meanwhile, mitophagy ensures mitochondrial health and functional activity by marking and removing damaged mitochondria through the actions of PTEN-induced kinase 1 (PINK-1) and parkin genes (Parkin) (65, 66). Mitochondrial dynamics play a critical role in regulating morphology and energy homeostasis (67). Mitofusins (Mfn-1 and Mfn-2) and optic atrophy protein 1 (OPA-1) act as effector proteins for mitochondrial fusion (68), mediating the fusion of the outer mitochondrial membrane (OMM) and inner mitochondrial membrane (IMM), respectively, to form larger mitochondrial networks (69). In contrast, dynamin-related protein 1 (Drp-1) and mitochondrial fission factor 1 (Fis1) facilitate mitochondrial fission, aiding in the removal of damaged mitochondria (70). These processes ensure the uniform distribution of energy while maintaining intracellular quality control.

Existing studies have demonstrated that PM_2.5_ exposure can lead to alterations in skeletal muscle mitochondrial morphology, such as cristae loss and outer membrane damage (24, 71, 72). Further studies have revealed that the extent of damage to skeletal muscle mitochondrial morphology varies with different durations of PM_2.5_ exposure. A study on short-term PM_2.5_ exposure reported that male mice exposed to PM_2.5_ (50.1 ± 8.1 μg/m^3^) three times within 1 week exhibited mitochondrial abnormalities, such as cristae swelling or cristae loss, in skeletal muscle. Muscle samples were processed 48 h after the final exposure. Additionally, a decrease in the expression of the mitochondrial outer membrane fusion protein Mfn1 was observed, while no significant changes were detected in other proteins involved in mitochondrial fission and fusion (24).

Another short-term PM_2.5_ exposure study (50.9 ± 10.4 μg/m^3^), involving continuous exposure for 5 days with a follow-up period, revealed sex-dependent effects on mitochondrial morphology. In male mice, 1 month after PM_2.5_ exposure, mitochondrial outer membrane damage was observed, accompanied by increased expression of mitochondrial fusion proteins (Mfn-1 and Mfn-2). In contrast, female mice exhibited more severe outer membrane damage, yet no changes were detected in mitochondrial dynamics-related factors. By 3 months post-exposure, mitochondrial morphology in both male and female mice showed recovery, and mitochondrial dynamics stabilized. Interestingly, at 15 months post-exposure, male mice exhibited signs of ongoing recovery, with increased expression of the fission protein Fis-1, while other dynamics-related factors remained stable. Conversely, female mice displayed smaller mitochondria under transmission electron microscopy (TEM) observation, decreased expression of the fission protein Drp-1, and a significant increase in Fis-1 expression. These changes suggested excessive mitochondrial fission. Additionally, among fusion proteins, only OPA-1 expression increased. These findings indicate that female mice might remain in a state of sustained mitochondrial damage (71).

In an experimental study of long-term PM_2.5_ exposure (71.20 ± 45.01 μg/m^3^), a 4-month exposure led to compromised mitochondrial membrane integrity in skeletal muscle, mild cristae loss, small vacuoles, and slight expansion of the sarcoplasmic reticulum. Over time, after 6 months of exposure, mitochondrial damage worsened, characterized by the presence of numerous mitochondrial vacuoles. Interestingly, in terms of mitochondrial dynamics, both fusion and fission protein expression showed significant increases compared to the control group (72). Compared to short-term exposure studies, the increased expression of fusion proteins Mfn-1, Mfn-2, and OPA-1 may represent a compensatory mechanism in response to the elevated expression of fission proteins (72).

**Figure 1:**
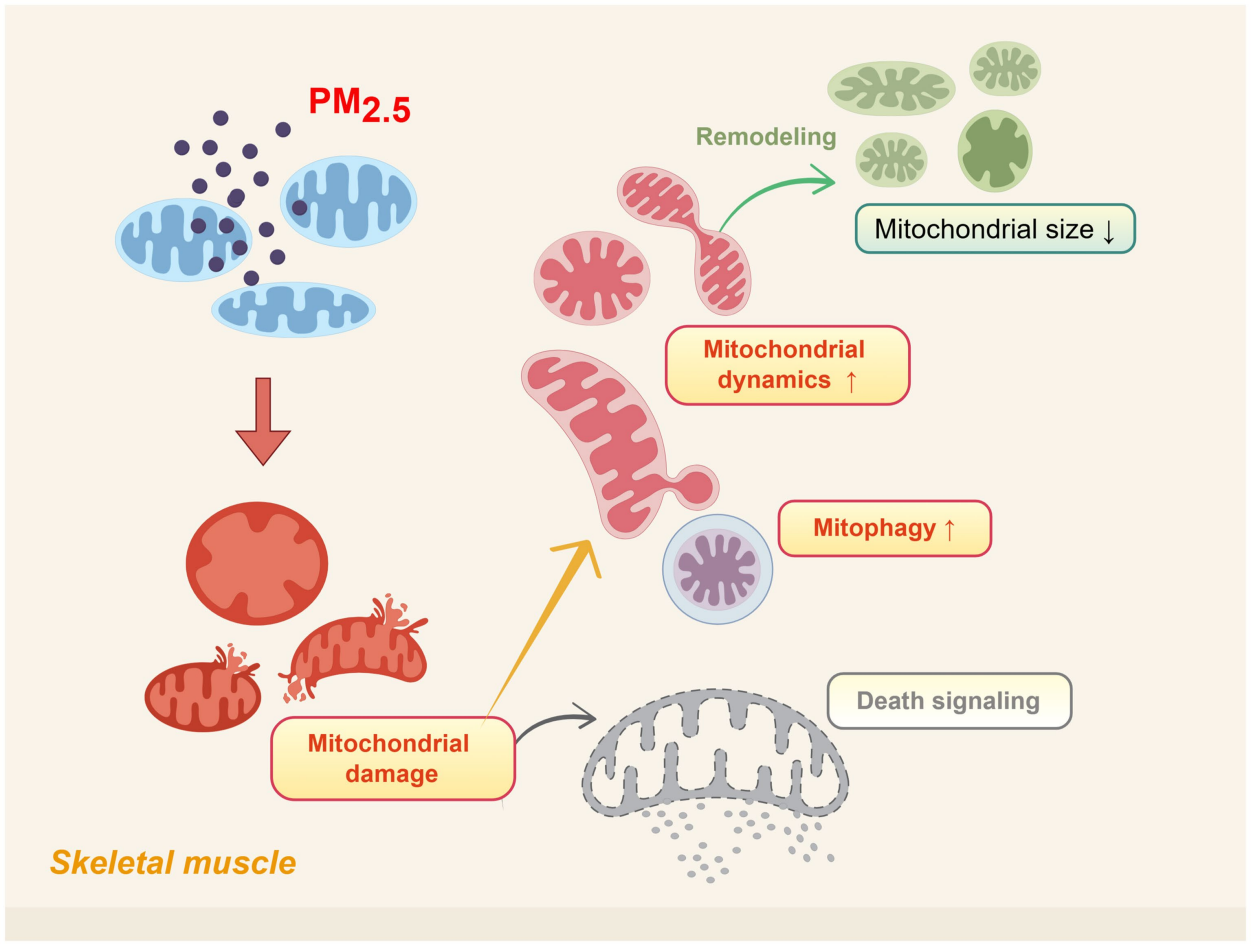
Schematic representation of skeletal muscle mitochondrial morphological damage induced by PM_2.5_ exposure. Including alterations in cristae structure, outer/inner membrane integrity, and increased mitochondrial fission activity. Created using Figdraw (ID: USTTAec5f8).

The above studies indicate that although most mitochondria survive after PM_2.5_ exposure, they fail to fully recover to their original state (Figure 1). The dose and duration of PM_2.5_ exposure may be positively correlated with the severity of mitochondrial damage (73). Higher concentrations or prolonged exposure can exacerbate the structural disruption of mitochondrial morphology (74). Notably, mitochondrial fusion and fission proteins may exhibit adaptive feedback mechanisms to maintain the balance of mitochondrial dynamics (71, 72, 75). Moreover, based on sex differences, the effects of PM_2.5_ exposure on mitochondrial morphology and dynamics exhibit significant variability (71).

It is well established that mitochondrial dynamics—such as number, size, and volume—are regulated through the coordinated processes of mitochondrial biogenesis and mitophagy (76). However, exposure to PM_2.5_ may disrupt this dynamic balance. PM_2.5_ exposure inhibits the SIRT-1/AMPKα/PGC-1α/NRF-1 signaling pathway in skeletal muscle, leading to a reduction in mitochondrial DNA copy number (72), a key marker of mitochondrial biogenesis (77). This suppression weakens the capacity for mitochondrial growth and proliferation (78). PM_2.5_ exposure has been shown to activate autophagy pathways (24). Although the precise mechanisms remain unclear, PINK-1, a mitochondrial enzyme, plays a key role when mitochondrial membrane damage occurs. PINK-1 accumulates on the outer membrane of damaged mitochondria, recruiting Parkin to initiate the autophagic pathway, thereby promoting ubiquitination (79, 80). This process facilitates the clearance of damaged mitochondria, helping to maintain mitochondrial quality within the cell (81). When mitochondrial biogenesis is impaired, the loss of damaged mitochondria cannot be compensated by the generation of new ones. This disruption of dynamic balance may lead to a reduction in the size of the remaining mitochondria or even a decrease in their total number within the cell (82).

### 2.2 Effects of PM_2.5_ exposure on mitochondrial enzymes

Skeletal muscle mitochondria produce ATP through three enzymatic pathways, fatty acid beta oxidation, the TCA cycle, and the ETC, constituting a chain reaction of oxidative metabolism (83, 84). Fatty acid β-oxidation primarily occurs within mitochondria (85). Prior to this process, the expression of carnitine palmitoyltransferase 1 (CPT1) is regulated by peroxisome proliferator-activated receptor α (PPARα) (86, 87). CPT1 catalyzes the transfer of long-chain fatty acids from coenzyme A to carnitine, enabling fatty acids to be transported into the mitochondria in the form of carnitine esters for β-oxidation (88), thereby generating substrates (acetyl-CoA) required for the TCA cycle (89). Subsequently, acetyl-CoA enters the TCA cycle, where it combines with oxaloacetate to form citrate under the catalytic action of citrate synthase (90). This marks the formal entry of acetyl-CoA into the TCA cycle, during which a series of enzymatic reactions generate key electron donors (NADH and FADH2) (37). These electrons are transferred through complexes I to IV of the electron transport chain, where they combine with oxygen to form water (91), ultimately driving ATP synthesis via complex V (ATP synthase) (92). This chain reaction ensures a stable energy supply for skeletal muscle metabolism.

A substantial body of research has demonstrated that exposure to PM_2.5_ in tissues such as the heart and liver suppresses the expression of PPARα and CPT1, thereby hindering the transport of fatty acids into mitochondria for β-oxidation (93, 94). Similar to the heart and liver, skeletal muscle also exhibits suppressed PPARα expression following PM_2.5_ exposure (71). However, long-term observations after PM_2.5_ exposure reveal that the damage to mitochondrial fatty acid β-oxidation is persistent and varies slightly depending on gender differences (71). One month after PM_2.5_ exposure, a decrease in the expression of PPARα and long-chain acyl-CoA dehydrogenase (LCAD) was observed in male mice, whereas no similar changes were detected in female mice (71). The reduction in PPARα expression may impair the efficiency of fatty acid transport into mitochondria (95, 96). Meanwhile, LCAD, a key enzyme in mitochondrial fatty acid β-oxidation (97), plays a crucial role in the breakdown of fatty acids during this process. Its decreased expression hinders the metabolic breakdown of fatty acids, slows down the β-oxidation pathway, and suppresses this critical mitochondrial energy supply mechanism (98, 99). At 3 months, the expression levels of PPARα and LCAD in male mice tended to return to normal, whereas female mice exhibited a decrease in PPARα expression (71). This may suggest that the damage induced by PM_2.5_ exposure on mitochondria could persist even after exposure has ceased (100). After 15 months of PM_2.5_ exposure, when the mice entered the aging phase, the expression of mitochondrial fatty acid β-oxidation proteins in male mice returned to normal. In contrast, in female mice, the phosphorylation of acetyl-CoA carboxylase (ACC) was suppressed (71). When ACC phosphorylation is suppressed, ACC activity increases, leading to more acetyl-CoA being converted into malonyl-CoA for fatty acid synthesis. The reduction in acetyl-CoA availability may compromise the efficiency of the TCA cycle (101).

Although PM_2.5_ exposure disrupts fatty acid β-oxidation and, through a chain reaction, further impacts oxidative phosphorylation (102). Nevertheless, the impact of PM_2.5_ exposure on oxidative phosphorylation is not solely related to fatty acid metabolism but is also mediated by abnormalities in energy metabolism caused by disruptions in enzymes within the TCA cycle. This is primarily reflected in the decreased expression of succinate dehydrogenase subunit B (SDHB) (24), a key enzyme linking the TCA cycle and the ETC (103). When PM_2.5_ exposure leads to a reduction in the expression of enzymes in the TCA cycle, the central role of the TCA cycle means that any damage to it could significantly impair the efficiency of oxidative phosphorylation (104). However, long-term observations of skeletal muscle mitochondria following PM_2.5_ exposure reveal that the effects vary by gender. One month after exposure, differences were observed in the expression of electron transport chain complexes: male mice exhibited reduced expression of mitochondrial complexes I, II, and IV, whereas female mice showed suppressed expression of complexes I–IV (71). This may be related to the morphological damage caused during the early stages of PM_2.5_ exposure, where severe structural damage leads to a significant decline in the expression of complexes housed within the mitochondrial cristae (105). By 3 months post-exposure, the expression of succinate ubiquinone oxidoreductase (SUO) and cytochrome c oxidase subunit 4 (COX-4) increased in male mice, while female mice returned to baseline levels. At 15 months, as the mice entered the aging phase, the electron transport chain expression in male mice tended to normalize, whereas female mice exhibited an increase in complex V expression (71). These effects highlight the long-term impact of PM_2.5_ exposure and its gender-specific differences, suggesting that mitochondrial damage caused by PM_2.5_ exposure may be lifelong. Future research should focus on the influence of PM_2.5_ exposure on aging-related metabolic changes.

### 2.3 Effects of PM_2.5_ exposure on mitochondrial function

Current studies often define mitochondrial functional damage through the observation of mitochondrial morphological abnormalities using transmission electron microscopy (TEM) (106), as well as through the evaluation of mitochondrial protein expression levels (such as quantity and overall protein abundance) (107). In fact, these indicators can only indirectly reflect the integrity of mitochondrial function. The activity of mitochondrial proteins, which reflects the efficiency of their functional execution, provides a more precise measure of mitochondrial function compared to expression levels (108). Therefore, the evaluation of mitochondrial function should focus on functional assessments, such as complex activity (108), reactive oxygen species (ROS) levels (109), respiration rate (110), and membrane potential (ΔΨm) (111), which are critical indicators.

Mitochondria are the primary site of ROS production within cells (112, 113). During the operation of the electron transport chain, partial electron leakage may occur during electron transfer from Complexes I and III (114, 115), leading to the formation of superoxide (O₂^−^) through interaction with molecular oxygen. This superoxide is subsequently dismutated into hydrogen peroxide (H₂O₂) by mitochondrial superoxide dismutase (SOD-2) (116). Hydrogen peroxide can be decomposed into water and oxygen under the action of glutathione peroxidase (GPx) using reduced glutathione (GSH), thereby mitigating oxidative damage (117, 118). ROS exhibit a dual role, under physiological conditions, the levels of ROS generation and antioxidant capacity are maintained in a state of equilibrium (119). Conversely, excessive accumulation of ROS or insufficient clearance can result in oxidative stress (120), and oxidative stress in skeletal muscle may be one of the key determinants of mitochondrial dysfunction (121).

In general, directly measuring intracellular ROS levels presents certain challenges. Most studies instead utilize the detection of lipid peroxidation byproduct malondialdehyde (MDA) as a surrogate marker (122, 123), MDA is a marker of lipid peroxidation and has the potential to induce cytotoxicity and cellular stress (124). It indirectly reflects intracellular ROS levels and the oxidative stress state through the measurement of thiobarbituric acid reactive substances (TBARS) (125). Recent studies have demonstrated that exposure to PM_2.5_ increases levels of MDA, a byproduct of lipid peroxidation, and hydrogen peroxide (H₂O₂) in skeletal muscle, leading to oxidative stress responses (126). After 15 months of PM_2.5_ exposure, the expression of TBARS in skeletal muscle remains at a high level, exacerbating oxidative stress responses in skeletal muscle (71).

In fact, mitochondria possess an antioxidant enzyme system capable of scavenging reactive oxygen species and maintaining redox balance (127). PM_2.5_ exposure suppresses the expression of superoxide dismutase 2 (SOD-2) in skeletal muscle (126). The downregulation of antioxidant enzyme expression disrupts the redox balance in the body (128), leading to excessive accumulation of ROS within mitochondria. This persistent oxidative stress response reduces the expression of mitochondrial DNA (mtDNA) (42). Interestingly, it is an established fact that exposure to PM_2.5_ exacerbates oxidative stress responses in skeletal muscle mitochondria (71, 72). However, the expression of antioxidant enzymes shows variability. In another study on short-term PM_2.5_ exposure, an increase in SOD-2 levels was observed (24). Although the expression of SOD-2 in mitochondria varies, including compensatory mechanisms associated with SOD-2 upregulation, it is evident that PM_2.5_ exposure disrupts the homeostasis of antioxidant enzymes. This disruption leads to ROS accumulation, resulting in sustained oxidative damage and subsequently contributing to mitochondrial dysfunction (129).

**Figure 2:**
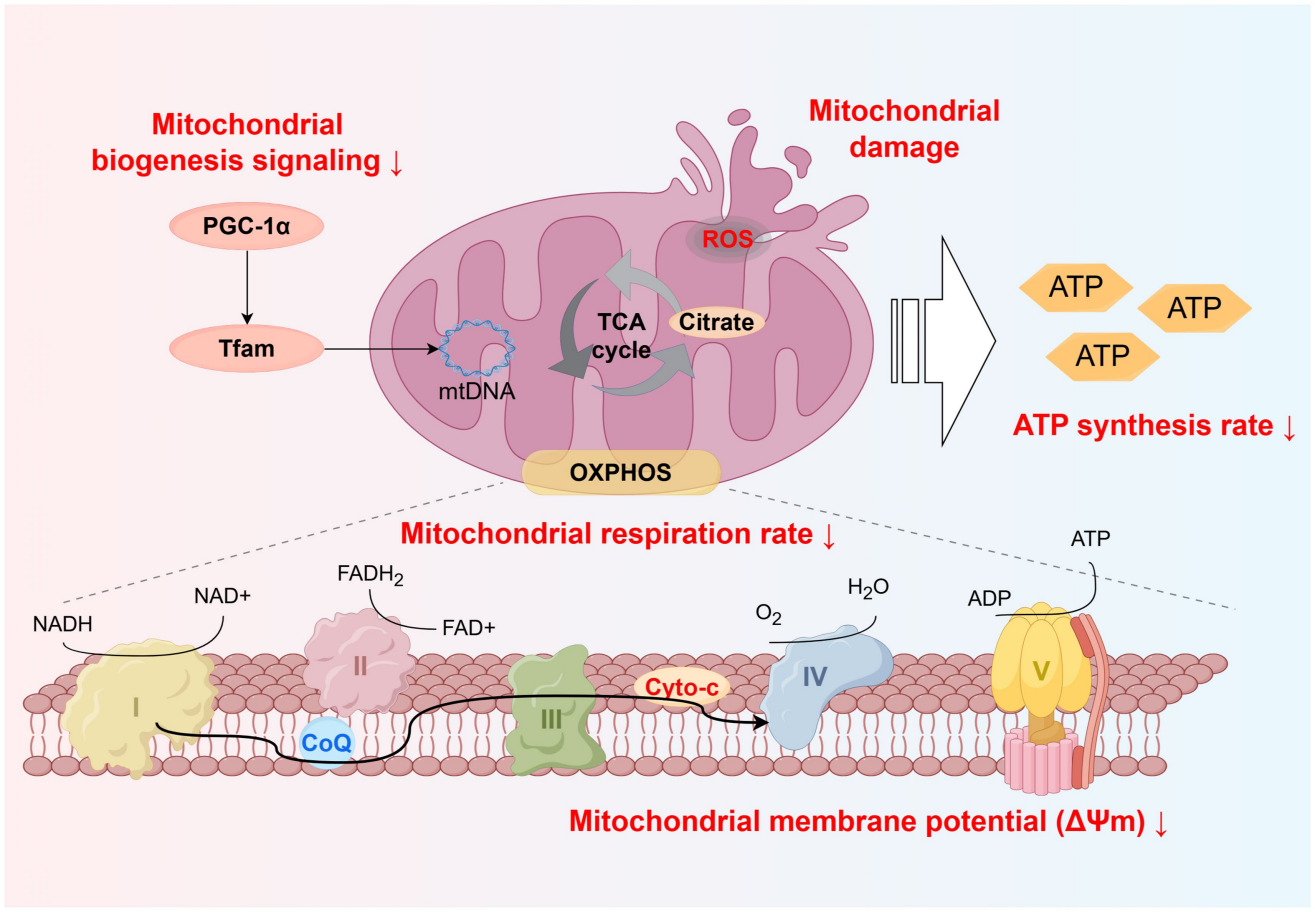
Schematic representation of skeletal muscle mitochondrial dysfunction induced by PM_2.5_ exposure. Including disrupted mitochondrial biogenesis signaling, increased ROS production, and reduced electron transport efficiency, leading to decreased mitochondrial respiration rate, membrane potential, and ATP synthesis. Created using Figdraw (ID: WSTWS9999d).

Notably, PM_2.5_ exposure can induce mitochondrial dysfunction by inhibiting mitochondrial enzyme activity (130), including a reduction in citrate synthase activity and a decline in overall cytochrome c oxidase (COX) activity (42, 126). Citrate synthase activity is commonly used as a marker of skeletal muscle aerobic capacity and mitochondrial density (131). A study on pregnant mice exposed to PM_2.5_ demonstrated that the reduction in citrate synthase activity leads to a decline in skeletal muscle oxidative capacity and a decrease in mitochondrial DNA content (42). Critically, citrate synthase is a key enzyme in the TCA cycle. A decline in citrate synthase activity may reduce the efficiency of citrate production from acetyl-CoA and oxaloacetate, thereby impairing overall TCA cycle efficiency (37). This disruption leads to a decreased generation of reducing equivalents (NADH and FADH₂), limiting electron input into the ETC (132). The mitochondrial Complexes I–V play critical roles in ATP synthesis (133), and their activity is closely associated with the efficiency of mitochondrial ATP production (134). Notably, a decline in mitochondrial cytochrome c oxidase (mtCOX) activity can lead to reduced oxidative capacity in skeletal muscle mitochondria (135). PM_2.5_ exposure can continuously impair mitochondrial functional integrity through a cascade of effects, including abnormalities in the antioxidant system, ROS accumulation, and decreased mitochondrial enzyme activity (Figure 2).

## 3 The impact of PM_2.5_-induced mitochondrial dysfunction on the skeletal muscle system

PM_2.5_-induced mitochondrial dysfunction has a variety of damaging effects on skeletal muscle, including oxidative stress, DNA damage, satellite stem cell senescence, muscle atrophy, and dysfunction of glycolipid metabolism. Table 2 summarizes studies on the effects of PM_2.5_ exposure on skeletal muscle system.

**Table 2:** Additional effects of PM_2.5_ exposure on skeletal muscle.

Studies	Treatment (PM)	Key findings	References
4-week-old mouse	6 h/day, 5 days/week, 10 months (Ohio Air Pollution Exposure System)	Insulin resistance increased, while the level of AKT phosphorylation in skeletal muscle was decreased	Xu et al. (181)
18-week-old wildtype and CCR2^−/−^ mice	6 h/day, 5 days/week, 17 weeks (Ohio Air Pollution Exposure System)	The GLUT-4 expression level in skeletal muscle was decreased	Liu et al. (161)
Human (older adult)	Cross-sectional survey	Long-term PM_2.5_ exposure is associated with decreased skeletal muscle mass and increased body fat mass in the older adult	Chen et al. (146)
Human	Cox proportional hazard regression models were applied to investigate the associations between pollutants and sarcopenia	Air pollution exposure elevated the risk of developing sarcopenia and related manifestations in a dose-dependent manner	Cai et al. (182)
8-week-old mouse	100 μg/m^3^, 1.5 h/day, 7 days (atmospheric source PM_2.5_)	MnSOD level, GSH/GSSG ratio and COX activity were significantly decreased. MDA and H_2_O_2_ increase causes oxidative stress	Park et al. (126)
4-week-old mouse	50.9 ± 10.4 μg/m^3^, 2 h/day, 5 days (atmospherically relevant artificial PM_2.5_)	Impaired development of muscle fibers, senescence of satellite cells, and sex-related lipid metabolism disorders in aging	Liu et al. (71)

### 3.1 Skeletal muscle oxidative stress and DNA damage

Oxidative stress can be regarded as a critical factor in PM_2.5_ exposure-induced mitochondrial dysfunction (71, 72, 126). Under excessive oxidative stress, skeletal muscle proteins undergo degradation (136), and damage is inflicted on the genetic material (DNA) of cells (137). Notably, the significant elevation of 8-hydroxy-2′-deoxyguanosine (8-OHdG) levels is recognized as a critical marker of DNA oxidative damage (138). PM_2.5_ exposure activates the expression of 8-OHdG, leading to endogenous oxidative DNA damage in the body (139, 140). Mitochondrial DNA (mtDNA) is considered more susceptible to oxidative DNA damage (141), and the accumulation of damage to both mitochondrial and nuclear DNA is believed to ultimately impair cellular function, leading to the loss of muscle cells (142). An experimental study found that in animal models exposed to PM_2.5_, the apoptosis rate of skeletal muscle cells significantly increased, accompanied by a reduction in muscle fibers (71). The cumulative effects of DNA damage have a significant impact on the regenerative capacity of skeletal muscle (143). As the core of muscle repair, skeletal muscle satellite cells rely on DNA stability for their proliferation and differentiation capacity (144). DNA damage can inhibit the proliferation and differentiation functions of skeletal muscle stem cells, thereby slowing the repair of muscle fibers and exacerbating the process of muscle aging (145).

### 3.2 Skeletal muscle maintenance and regeneration

To date, cross-sectional studies have shown that for every 1.41 μg/m^3^ increase in PM_2.5_ concentration, skeletal muscle mass decreases by 0.4 kg (146). Additionally, for every 1 μg/m^3^ increase in PM_2.5_, the risk of sarcopenia increases by 11.1% (147), and every 10 μg/m^3^ increase in PM_2.5_ may result in a 0.7 kg reduction in grip strength (148). The above evidence indicates that PM_2.5_ exposure can lead to muscle atrophy and a decline in muscle strength.

An animal experimental study revealed that PM_2.5_ exposure can induce stem cell senescence and regenerative dysfunction, resulting in severe damage to muscle fibers. Moreover, the extent of the damage varies depending on age and gender (71). Specifically, PM_2.5_ exposure led to a significant reduction in the number of muscle fibers in juvenile male mice and decreased the expression of Myogenin (71). Myogenin, a critical transcription factor essential for muscle differentiation and regeneration (149), is suppressed, which weakens the regenerative capacity of muscle cells (150). In addition, PM_2.5_ exposure significantly upregulated the Bax/Bcl-2 ratio in juvenile male mice (71). The excessive expression of the Bax/Bcl-2 ratio is associated with increased muscle cell apoptosis (151). Exposure to PM_2.5_ during the juvenile period resulted in simultaneous muscle cell apoptosis and myostatin inhibition, further reducing the number of muscle fibers; although muscle fibers in adult male mice showed partial recovery, they failed to return to their original state, possibly due to the normalization of myostatin levels, but the recovery process remained slow with low regenerative efficiency owing to continued muscle cell apoptosis (71). In contrast, adult female mice exhibited significant muscle fiber damage at this stage, accompanied by simultaneous reductions in the expression of Myostatin and Myogenin (71). This disruption in the balance of skeletal muscle growth and differentiation likely further impairs muscle fiber regenerative capacity, increasing the risk of muscle atrophy (152, 153). Upon entering the aging stage, male mice exhibited persistent muscle fiber damage, which reflected a further decline in regenerative capacity, and although Myogenin expression was significantly elevated, indicating a compensatory attempt at regeneration, the repair outcomes remained limited (71). Interestingly, the number of muscle fibers in female mice returned to normal, which may be associated with the significant upregulation of Myogenin and Pax-7 expression (154). PM_2.5_ exposure also activated the expression of senescence markers β-galactosidase (β-Gal) and cyclin-dependent kinase inhibitor 2A (p16) in female mice, accelerating the aging of muscle stem cells (71).

In summary, the effects of PM_2.5_ exposure vary across different age groups, with juvenile male mice being more sensitive to PM_2.5_ exposure, while skeletal muscle fibers in the older adult stage experience the most severe damage. The muscle fiber damage caused by PM_2.5_ exposure is persistent, potentially linked to mitochondrial damage induced by PM_2.5_, which disrupts the energy supply required for skeletal muscle growth and regeneration. This process accelerates skeletal muscle atrophy, manifested as muscle fiber shrinkage and decreased muscle strength, thereby increasing the risk of conditions such as sarcopenia.

### 3.3 Skeletal muscle metabolic function

Skeletal muscle is a critical organ for glucose and fatty acid metabolism (155, 156). Mitochondrial dysfunction induced by PM_2.5_ exposure compromises ATP production, failing to meet the metabolic demands of cells (36). Consequently, cells may respond through adaptive changes in metabolic pathways, such as glucose and lipid metabolism (157, 158).

Skeletal muscle is the primary organ where insulin-mediated glucose uptake occurs through glucose transporter 4 (GLUT4) (159). A short-term PM_2.5_ exposure study demonstrated that although mitochondrial oxidative phosphorylation capacity in skeletal muscle declines, it compensates for the energy deficit by upregulating the expression of hexokinase 2 (HK2) (24). HK2, a key enzyme in the glycolytic pathway, enhances glucose uptake and utilization to sustain energy supply and adapt to metabolic stress (160). It has been reported that PM_2.5_ exposure reduces the expression of GLUT4 (71, 161), and this reduction impairs the ability of skeletal muscle to uptake glucose, leading to disruptions in glucose metabolism within skeletal muscle and the development of insulin resistance (162, 163). However, under the long-term effects of PM_2.5_ exposure, GLUT4 expression gradually increases over time, potentially linked to the decline in OXPHOS efficiency caused by mitochondrial dysfunction. This shift in cellular metabolism may progressively favor a glycolysis-dominant energy production mode, with upregulated GLUT4 expression enhancing glucose dependency (71).

Skeletal muscle is a crucial site for fatty acid metabolism (164). PM_2.5_ exposure affects the expression of lipid metabolic enzymes in skeletal muscle, with the downregulation of peroxisome proliferator-activated receptor alpha (PPARα) and long-chain acyl-CoA dehydrogenase (LCAD), leading to reduced fatty acid utilization and gradual lipid accumulation within the muscle (71). Abnormal lipid metabolism is more pronounced in females than in males after PM_2.5_ exposure, with females being more severely affected, which can lead to conditions such as obesity (71).

Changes in metabolic enzymes within skeletal muscle caused by mitochondrial dysfunction disrupt the homeostasis of glucose and lipid metabolism, impairing the metabolic function of skeletal muscle. Both glucose homeostasis imbalance and abnormal lipid metabolism are closely associated with insulin resistance (163, 165). This metabolic dysregulation not only exacerbates the energy metabolism burden on the body but also provides a critical pathological basis for the development of diabetes and related metabolic syndromes.

## 4 Regulatory effect of exercise on PM_2.5_-induced mitochondrial dysfunction

Exercise, as a non-pharmacological intervention, has been shown to mitigate the health risks associated with exposure to PM_2.5_ (126, 166). Studies have shown that long-term moderate exercise under conditions of low PM_2.5_ concentrations is beneficial to health (72). The Table 3 summarizes studies on the moderating effect of exercise on the damage caused by PM_2.5_ exposure.

**Table 3:** The moderating effect of exercise on the damage caused by PM_2.5_ exposure.

Studies	Treatment (exercise)	Key findings	References
Human	Low, moderate or high physical activity levels at living atmosphere	Habitual physical activity was associated with statistically significant lower markers of systemic inflammation across different levels of PM_2.5_	Zhang et al. (170)
8-week-old rat	Treadmill 20–50 min/time, 5 days/week with a moderate intensity of 70% for 90 days	Exercise can increase the activity of the SOD in the gastrocnemius muscle and reduce the level of TBARS, thereby reducing the oxidative stress	Marmett et al. (169)
16-month-old rat	Treadmill-trained for 8 weeks (65–75% VO2max for 30 min every other day)	Aerobic pre-exercise had protective effects on lung injury and reduced vulnerability to inflammation induced by PM_2.5_ exposure, possibly through the TLR4/NF-κB signaling pathways mediated by the extracellular-to-intracellular HSP70 ratio	Qin et al. (166)
8-week-old mouse	Treadmill exercise for 60 min at 20 m/min with a 5-degree uphill incline once a day for 1 week	PM-induced adverse effects on the lung tissue are not exacerbated by exercise-induced PM hyperventilation but rather has a protective effect	So et al. (167)
1/12-month-old mouse	Treadmill for 40 min at 8–10 min/min, 5 times/week for 8 weeks	Aerobic exercise training led to significantly lower 8-OHdG, MDA, IL-1β, IL-6, and TNF-α levels and significantly higher SOD and CAT activities in both age groups receiving exercise training	Cho et al. (140)
8-week-old mouse	Exercise was performed for 90 min per day for 7 days, the treadmill being set at 20 m/min on a 5-degree uphill slope	PM has adverse effects concerning both oxidative stress and inflammatory responses in skeletal muscle and mitochondria, both at rest and during exercise	Park et al. (126)
16-week-old mouse	12 weeks of training by treadmill (0% incline, 10–15 m/min, 60 min/day, 4 times/week)	Increased aerobic fitness through endurance training can mitigate PM_2.5_-induced mitochondrial damage	Liu et al. (24)
7-week-old mouse	2/4/6 months treadmill exercise at a speed of 12 m/min and 25% incline for 1 h	PM_2.5_ may impact mitochondrial biogenesis and dynamics, which further lead to IR, glucose and lipid disorders. However, exercise might alleviate the damages caused by PM_2.5_ exposure	Fan et al. (72)

### 4.1 Long-term endurance exercise can prevent PM_2.5_ exposure-induced mitochondrial dysfunction

Regular aerobic exercise can mitigate PM_2.5_-induced damage and oxidative stress by activating the SIRT-1/AMPKα/PGC1-α/NRF-1 signaling pathway (72). As a key regulator of mitochondrial biogenesis (59), PGC-1α enhances mtDNA replication and transcription by upregulating downstream factors such as mTFA, counteracting the suppression of mitochondrial biogenesis caused by PM_2.5_ exposure. This significantly improves the reduction in mitochondrial number and size induced by PM_2.5_ exposure, with the beneficial effects becoming more pronounced with prolonged exercise duration (72). In addition, exercise significantly reduced PM_2.5_-induced mitochondrial damage levels, representing another mechanism for preventing PM_2.5_-induced damage (167).

**Figure 3:**
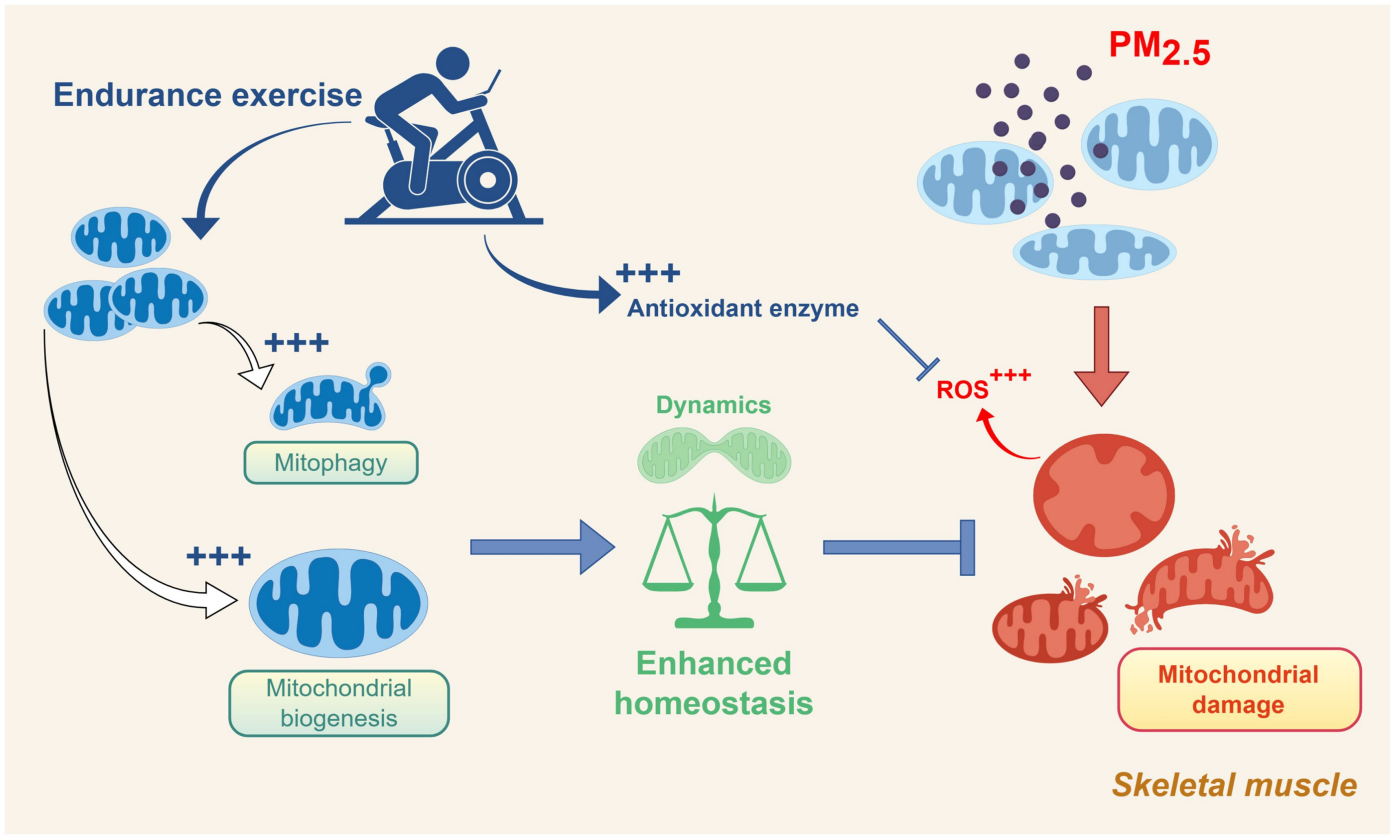
Schematic representation of the protective role of endurance exercise against skeletal muscle mitochondrial dysfunction induced by PM_2.5_ exposure. Exercise promotes mitochondrial biogenesis, mitophagy, and antioxidant defense, enhancing mitochondrial homeostasis and counteracting PM_2.5_-induced oxidative stress and damage. Created using Figdraw (ID: PWSAW4e4ee).

PM_2.5_ exposure leads to mitochondrial morphological damage and dynamics disruption. Although long-term endurance training cannot completely prevent acute skeletal muscle mitochondrial damage caused by acute exposure to PM_2.5_ (50.1 ± 8.1 μg/m^3^, 2 h/day, 5 days), a higher level of exercise adaptation can promote repair and regeneration following the damage (24). While endurance training can maintenance mitochondrial morphology, this adaptation does not prevent mitochondrial morphological damage under PM_2.5_ exposure or immediately following exercise after exposure (24). However, the activation of mitochondrial autophagy levels induced by long-term endurance exercise results in a rapid response to clear damaged mitochondria following exposure to PM_2.5_ (24). Endurance exercise enhances mitochondrial dynamics by activating mitophagy and biogenesis, thereby reinforcing the mitochondrial homeostasis (Figure 3).

PM_2.5_ exposure induces mitochondrial dysfunction by inhibiting the expression and activity of mitochondrial respiratory chain complexes (71, 126). Long-term endurance exercise significantly enhances the expression of Complexes I, II, and IV, increasing their tolerance to oxidative damage caused by PM_2.5_ (24). Regular aerobic exercise further boosts the activity of Complex IV, thereby improving oxidative phosphorylation efficiency and reducing excessive ROS accumulation (126).

PM_2.5_-induced mitochondrial damage is often accompanied by a significant increase in ROS production, which subsequently triggers oxidative stress and inflammatory responses (167). Notably, endurance training enhances antioxidant capacity and alleviates inflammatory responses, which helps maintain health under conditions of PM_2.5_ exposure (168). Appropriate aerobic exercise can enhance the function of the body’s antioxidant system and increase the expression of superoxide dismutase (SOD) and catalase (140), with long-term regular aerobic exercise further improving SOD activity and reducing lipid peroxidation in skeletal muscle (169). In addition to the aforementioned effects, exercise can also mitigate systemic inflammatory responses by reducing inflammatory markers, such as leukocytes (170).

It is worth noting that PM_2.5_ (100 μg/m^3^, 1.5 h/day, 7 days) exposure during exercise can exacerbate oxidative stress responses in skeletal muscle, leading to more severe mitochondrial dysfunction (126). This is primarily because intense exercise in high-concentration environments increases respiratory volume, resulting in greater inhalation of PM_2.5_, which in turn triggers oxidative stress and inflammatory responses, adversely affecting skeletal muscle health (126).

The above results clarify the preventive and regulatory effects of long-term endurance exercise on PM_2.5_ exposure-induced skeletal muscle mitochondrial damage. The results consistently demonstrate the necessity of long-term endurance exercise, while also indicating that PM_2.5_ concentrations in the environment should not be too high during endurance exercise. When PM_2.5_ levels are higher, it is recommended to moderately reduce outdoor endurance exercise time to minimize PM_2.5_ intake. At the same time, awareness of using dust masks outdoors should be enhanced to avoid exposure to high concentrations. When PM_2.5_ concentrations persistently exceed the aforementioned minimum experimental concentration (50 μg/m^3^), it is recommended to relocate endurance exercises indoors and implement air purification measures (such as using air purifiers) to reduce indoor exposure levels. Therefore, appropriately arranging the intensity and location of endurance exercise may help reduce the potential damage to skeletal muscle mitochondria caused by PM_2.5_ exposure while enjoying the health benefits of exercise.

### 4.2 The preventive and treatment potential of low-intensity resistance exercise against PM_2.5_ exposure-induced skeletal muscle mitochondrial damage

The effects of exercise treatment in the current PM_2.5_ exposure models are all based on endurance exercise (treadmill) (Table 3). This may be due to the convenience of animal models for endurance exercise treatment and the significant specificity of endurance exercise in regulating mitochondrial function (171).

However, recent studies have shown that long-term low-intensity resistance exercise can also enhance mitochondrial biogenesis and regulate mitochondrial homeostasis (172). At the same time, low-intensity resistance training also has similar skeletal muscle protein synthesis gains to moderate- and high-intensity resistance training (173). Compared to endurance exercise, resistance training relies less on the aerobic oxidation function of mitochondria and mainly relies on the anaerobic glycolysis of muscle glycogen to rapidly provide ATP (29). Therefore, the level of ROS produced by mitochondria induced by resistance exercise stimulation is lower (174), while resistance exercise promotes the activation of lactate signaling, which also has multiple effects on the regulation of mitochondrial function (175). Thus, low-intensity resistance exercise may have a positive regulatory effect on skeletal muscle and mitochondria that differs from endurance exercise during periods of PM_2.5_ exposure or when PM_2.5_ exposure-induced mitochondrial damage occurs.

Moderate- to high-intensity resistance training causes extensive microdamage to skeletal muscle fibers and activates inflammation-related pathways (176). Moreover, the damage and remodeling process of the skeletal muscle system caused by moderate-to-high-intensity resistance exercise can induce sustained cellular stress and high ATP demand during the repair phase (176), which may result in more severe compound damage during periods of PM_2.5_ exposure.

In summary, low-intensity resistance exercise has the potential to prevent and regulate PM_2.5_ exposure-induced mitochondrial dysfunction and should be further investigated in future studies. On the other hand, high-intensity resistance training should be avoided when PM_2.5_ concentrations exceed the standard, even if training is conducted indoors.

### 4.3 Potential synergistic role of exercise combined with antioxidants

Antioxidants, as exogenous interventions, can effectively reduce the occurrence of oxidative stress (177). Although there are many types of antioxidants, and the mechanisms and targets of various antioxidants differ (177). However, studies have shown that multiple antioxidants have synergistic effects in alleviating oxidative stress while maintaining mitochondrial function (178, 179). Mitochondria-targeted antioxidant MitoQ can alleviate PM_2.5_-induced vascular fibrosis and related oxidative damage (178). Vitamin E and omega-3 fatty acids may reduce vascular endothelial cell inflammation and oxidative stress responses caused by exposure to PM_2.5_ (180). Although evidence linking skeletal muscle-targeted interventions to combined interventions remains limited, the effects of these independent interventions on other tissues provide important evidence for the development of synergistic strategies.

## 5 Limitations of the current research

Due to the current lack of human skeletal muscle targeting research in this field, the experimental studies included in this review are mainly based on rodent models. Although these models provide valuable insights into the mechanisms underlying PM_2.5_ exposure-induced skeletal muscle mitochondrial damage, their findings may have limitations in terms of applicability to humans. On the one hand, rodents and humans have certain differences in metabolic rate and movement adaptation patterns, which may lead to differences in their sensitivity and expression of stress responses and intervention effects caused by PM_2.5_. In addition, most animal studies use acute or high-dose exposure models, which differ from the long-term, low-dose exposure environment that humans encounter in real life.

In future studies, further exploration of population-based longitudinal observational and interventional studies should be conducted, combining real-life exposure scenarios to systematically assess the long-term effects of PM_2.5_ on human skeletal muscle mitochondrial function. And explore the dose–response relationship under PM_2.5_ exposure and the potential protective mechanisms under different types of exercise interventions. In addition, attention should be paid to the toxic effects of different components of PM_2.5_ and their specific impact on skeletal muscle damage.

## 6 Conclusion

Studies on the effects of PM_2.5_ exposure on skeletal muscle mitochondria have shown that exposure to PM_2.5_ induces significant alterations in mitochondrial morphology and disrupts the balance of mitochondrial dynamics, biogenesis, and autophagy processes. By inhibiting fatty acid β-oxidation and oxidative phosphorylation efficiency, PM_2.5_ exposure ultimately leads to a reduction in ATP production capacity and disruptions in energy metabolism. The oxidative stress response induced by PM_2.5_ exposure can reduce mitochondrial enzyme activity, exacerbating mitochondrial dysfunction. It may also cause DNA damage in skeletal muscle cells and disrupt the balance of skeletal muscle maintenance and regeneration. This accelerates muscle fiber atrophy and increases the risk of sarcopenia while profoundly affecting glucose and lipid metabolic homeostasis, further elevating the incidence of metabolic diseases. Although regular aerobic exercise has been shown to alleviate the negative effects of PM_2.5_ exposure by activating certain mitochondrial pathways, exercising in high-concentration PM_2.5_ environments may exacerbate oxidative damage, highlighting the double-edged nature of exercise interventions. When daily PM_2.5_ exceeds 50 μg/m^3^, endurance exercise should be moved indoors. In summary, future research should not only elucidate the molecular mechanisms underlying the detrimental effects of PM_2.5_ on mitochondrial function but also focus more on potential therapeutic intervention strategies, such as combined interventions involving exercise and antioxidants.
